# Fundus Autofluorescence and Spectral Domain OCT in Central Serous Chorioretinopathy

**DOI:** 10.1155/2011/706849

**Published:** 2011-08-08

**Authors:** Luiz Roisman, Daniel Lavinsky, Fernanda Magalhaes, Fabio Bom Aggio, Nilva Moraes, Jose A. Cardillo, Michel E. Farah

**Affiliations:** Department of Ophthalmology, Federal University of São Paulo, Paulista School of Medicine, 04025-011 São Paulo, SP, Brazil

## Abstract

*Background*. To describe the standard autofluorescence (FAF), the near infrared autofluorescence (NIA) and optical coherence tomography (OCT) patterns in central serous chorioretinopathy, correlating them with fluorescein angiography. *Methods*. Cross-sectional observational study, in which patients with at least seven months of CSC underwent ophthalmologic examination, fundus photography, FAF, NIA, fluorescein angiography (FA), and spectral-domain OCT. *Results*. Seventeen eyes of thirteen patients were included. The presentation features were a mottled hyperFAF in the detached area and areas with pigment mottling. NIA images showed areas of hyperNIA similar to FAF and localized areas of hypoNIA, which correlated with the points of leakage in the FA. OCT showed pigment epithelium detachment at the location of these hypoNIA spots. *Discussion*. FAF showed increased presence of fluorophores in the area of retinal detachment, which is believed to appear secondary to lipofuscin accumulation in the RPE or the presence of debris in the subretinal fluid. NIA has been related to the choroidal melanin content and there were areas of both increased and decreased NIA, which could be explained by damage ahead the retina, basically RPE and choroid. These findings, along with the PEDs found in the areas of hypoNIA, support the notion of a primary choroidal disease in CSC.

## 1. Introduction

Central serous chorioretinopathy (CSC) is an idiopathic syndrome of young to middle-aged adults, characterized by serous detachment of the neurosensory retina with focal and multifocal areas of leakage at the level of the retinal pigment epithelium (RPE), predominantly affecting the macular area [1]. Patients often complain of blurred central vision, micropsia, and metamorphopsia [2]. This idiopathic syndrome has been associated with systemic corticosteroid therapy [3] and emotional distress [4]. In most cases, CSC resolves spontaneously within six months, with good visual prognosis [5]. Prolonged and recurrent macular detachment in some cases, however, may cause degenerative changes in the subfoveal RPE and neurosensory retina with poor visual outcome [6].

The understanding of the physiopathology of CSC remains limited due to the lack of significant histopathologic studies. Investigational and diagnostic tools such as fluorescein and indocyanine green angiography have provided some insight into the mechanism of this disease [7]. Optical coherence tomography (OCT) has provided additional data about central macular detachments [8], development of retinal atrophy, and the correlation with visual acuity in resolved central serous chorioretinopathy [9]. Fundus autofluorescence (FAF) photography (488 nm) provides functional images of the fundus by employing the stimulated emission of light from endogenous fluorophores, the most significant being lipofuscin. In the case of RPE cells, the buildup of lipofuscin is related in large part to the phagocytosis of damaged photoreceptor outer segments and altered molecules retained within lysosomes, which eventually become lipofuscin [10–12]. Moreover, near infrared fundus autofluorescence (NIA) imaging (787 nm) is able to study the RPE, the choriocapillaris, and choroid, by determining melanin fluorescence [13]. 

The purpose of this study was to describe NIA and SD-OCT findings in CSC, correlating them with fluorescein angiography (FA). To our knowledge, this is the first study that investigates the correlation between the clinical findings and different imaging modalities, specially with NIA.

## 2. Materials and Methods

This is a retrospective, cross-sectional study were patients with CSC were included from September 2008 to May 2009 at the Federal University of Sao Paulo. This study was conducted in accordance with the Declaration of Helsinki and was approved by the ethics committee of the Federal University of Sao Paulo. All patients had experienced symptoms for at least 7 months and none of them had previous treatment.

Each patient underwent ophthalmologic examination, color fundus photography, FAF and NIA imaging, fluorescein angiography (FA), and HRA Spectralis spectral-domain OCT (SD-OCT) (Heidelberg Engineering, Heidelberg, Germany). The OCT protocol used was volume in the SD-OCT. The volume size was individualized for each patient. FAF, NIA, and FA imaging were performed with the Heidelberg Retina Angiograph 2 system (HRA2, Heidelberg, Germany). FAF and NIA images were recorded at 488 nm, using a barrier filter for detection of emitted light above 500 nm, and 787 nm, respectively. At least 5 single AF images of 512 × 512 pixels were acquired with each method. Several images were aligned and a mean image was calculated after detection and correction of eye movements using image analysis software.

Abnormal AF was defined as either increased or decreased fundus AF in comparison with background fundus AF [13], and classified as either hyperautofluorescence, hypo-autofluorescence, or mixed autofluorescence (no clear predominance).

## 3. Results

We included 17 eyes of 13 patients, eight males, and five females, presenting CSC with a mean time of complaint of 12.7 months, ranging from 7 months to 2 years, with age ranging from 26 to 53 years (mean age of 39.3 years). Two individuals had history of using steroids, but had stopped the use for at least 6 months. Fifteen patients had a relapsing desease and two were in the first episode.

Fluorescein angiography showed in 8 (47%) eyes focal leakage, in 1 (5.9%) multifocal leakage, in 4 (23.5%) multiple window defect with diffuse leakage, and in 4 (23.5%) no leakage. 

Ten eyes exhibited serous retinal detachment. Of those, 1 presented with multifocal leakage, 7 with focal leakage, and 2 with RPE changes and multiple windows defects. The detached area revealed hyperFAF in 7 (70%), mixedFAF in 2 (20%), and hypoFAF in 1 (10%). HypoNIA could be seen in 5 (50%), mixedNIA in 2 (20%), and hyperNIA in 3 (30%). 

The areas of window defects seen at FA in 8 eyes showed hyperFAF in 6 (75%), mixedFAF and hypoFAF in 1 eye each (12.5%). Concerning NIA of the window defects areas, we could find 5 eyes indicating hypoNIA (62.5%) and 3 mixedNIA (37.5%). 

The overall FAF images revealed hyperFAF in 12 (70.6%) eyes, hypoFAF in 3 (17.6%) eyes, and mixed FAF in 2 (11.8%) eyes. The overall NIA images presented hyperNIA in 4 (23.5%) eyes, hypoNIA in 10 (58.8%), and mixed NIA in 3 (17.6%) eyes. Twelve of thirteen eyes with leakage showed a hypoNIA exactly at the leaking spot. A hypoFAF was found in 3 eyes in the leakage site. The location of autofluorescence alterations were perimacular, following the leakage and the RPE damage sites (Table 1).

The Spectralis OCT images revealed 10 (58.8%) foveal neurosensory retinal detachment. The mean foveal retinal thickness was 332.6 *μ*m, ranging from 220 to 533 *μ*m. Pigment epithelium detachment (PED) in foveal and/or extrafoveal spots was identified in 11 of the 13 patients, in a total of 22 PEDs in 15 of 17 eyes. Twelve (54.5%) were foveals and ten extra foveals (45.4%). We could observe in 5 eyes a substantial RPE irregularity, characterized by RPE undulations, 2 of them without PED. 

**Figure 1:**
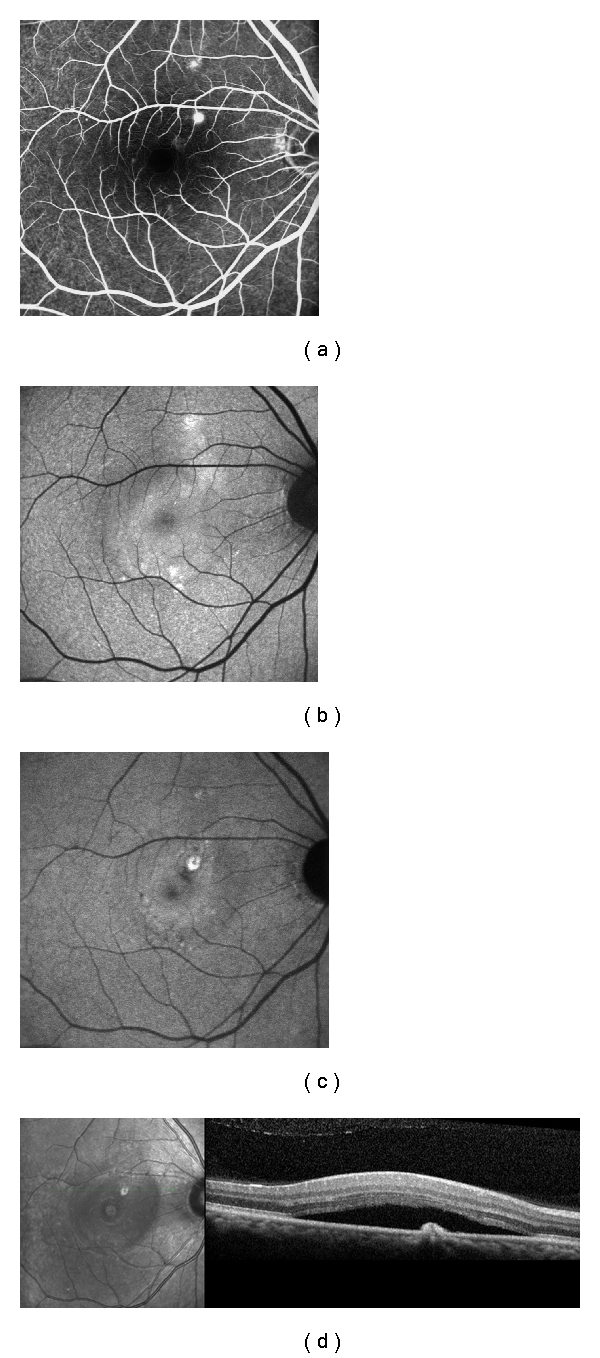
Patient with 12 months of history of CSC in the right eye. (a) Two dots spots of leakage in the angiography; (b) standard FAF, presenting with diffuse hyperFAF; (c) NIA, showing suprafoveal hypofluorescent point surrounded by a ring of hyperAF, corresponding exactly with the PEDs in (d); (d) SD-OCT exhibiting the PED and neurosensorial retinal detachment.

**Figure 2:**
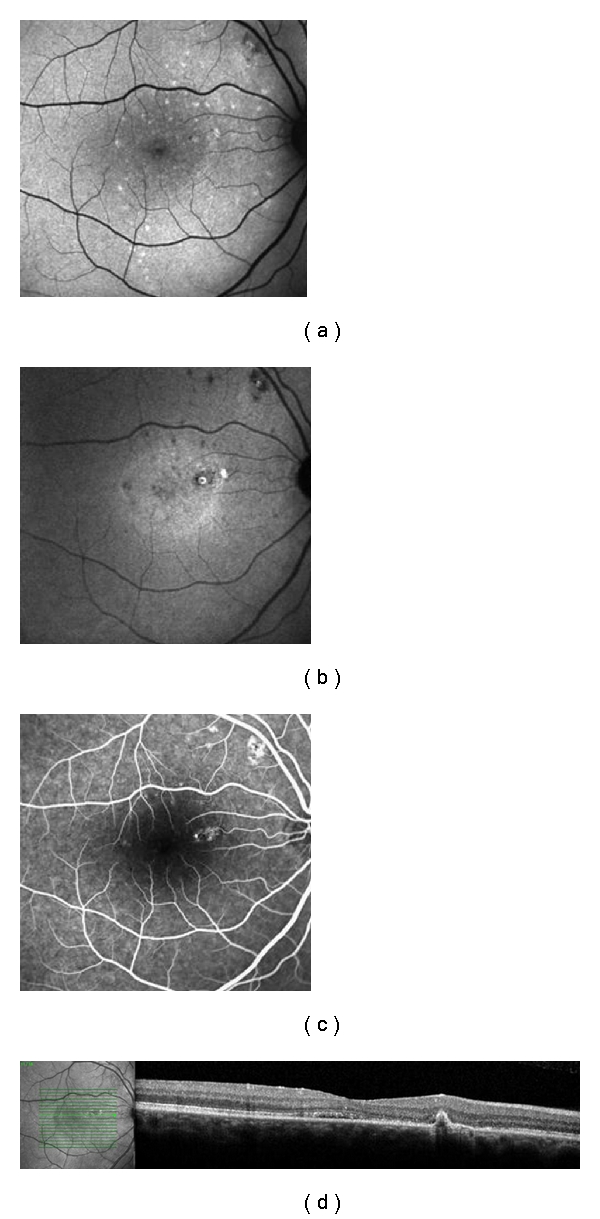
Patient with 9 months of history of CSC in the right eye. (a) Standard FAF, showing mottled hyperFAF spots; (b) NIA, presenting macular and supratemporal to the optic disk hypofluorescent points—the macular spot surrounded by a ring of hyperAF, corresponding exactly with the PED in (d); (c) spots of leakage in the angiography; (d) SD-OCT exhibiting the PED.

**Figure 3:**
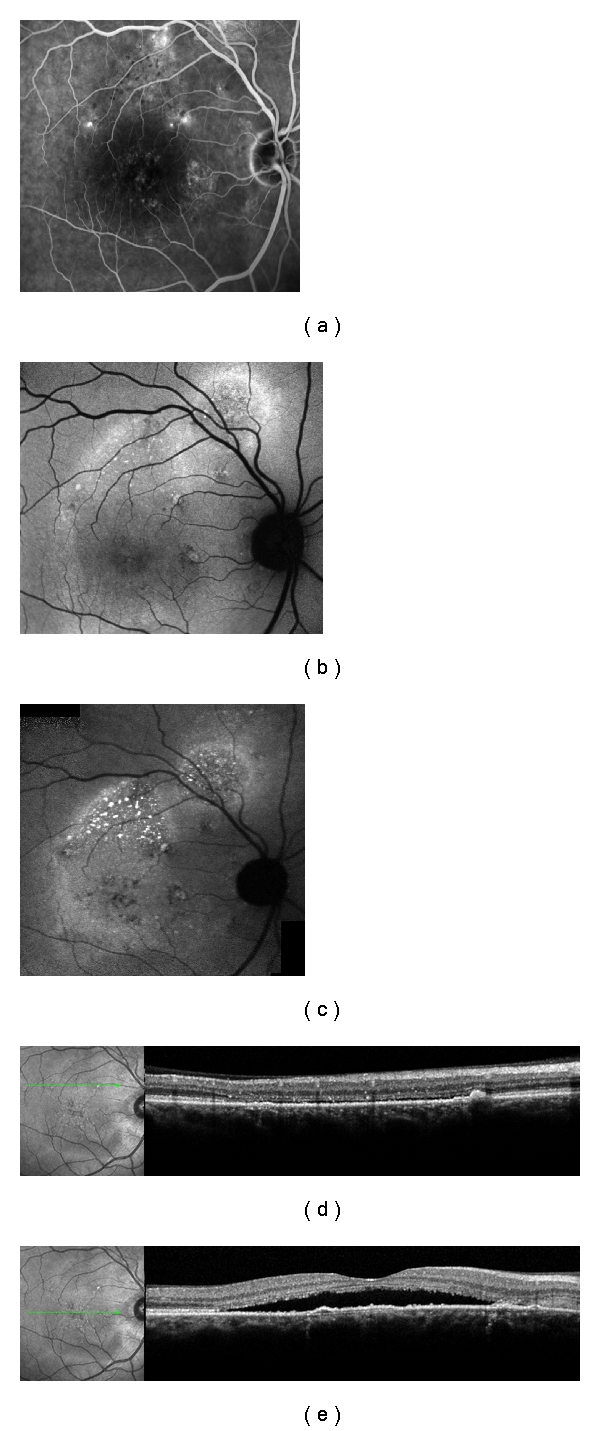
Patient with 18 months of history of CSC in the right eye. (a) dots spots of leakage in the angiography; (b) standard FAF, showing diffuse superior hyperFAF; (c) NIA, showing three hypofluorescent spots around the macula, some of them corresponding exactly with the PEDs in (d) and (e); (d) and (e) SD-OCT exhibiting the PEDs and neurosensorial retinal detachment.

A hyperNIA spot in the NIA images could be seen in 8 (36.4%) of the PEDs and in 14 (63.6%) corresponding to hypoNIA sites. In 7 (31.8% of all PEDs) of the 14 PEDs that corresponded to hypoNIA images, we could identify a hyperNIA “ring.” In 14 (63.6%) of the PEDs, we found that the PED corresponded exactly with the leakage spot detected in FA, and 7 (31.8%) PEDs corresponded to window defect areas in FA, while 1 (4.5%) PED did not correlate with FA alterations. The 14 PEDs that correlated with the point of leakage showed a hyperNIA point in 5 (35.7%) PEDs, hypoNIA with hyperNIA “ring” in 4 (28.6%), and hypoNIA without ring in 5 (35.7%) of the leakage PEDs (Figures 1, 2 and 3). 

## 4. Discussion

NIA is not a common used modality of the HRA2, but its importance when studying the choroid and outer retina had been described before [13]. Recently, Kellner et al. suggests that NIA is a reliable method for RPE evaluation in AMD [14]. It can be very helpful for conditions such as CSC, in which the choroid appears to be primarily involved. In the present study, it was shown that the mostly hypofluorescent spots on NIA images corresponded to pigment epithelium detachments on OCT and to leakage points on fluorescein angiography. The hypoNIRAF spot could identify the leakage points in 12 (70.6%) of the eyes and the ring of hyperNIA around these hypofluorescent spots found in 31.8% of all PEDs could represent “pooling” of RPE cells because of the PEDs or local RPE folding, but further studies are needed to confirm this hypothesis. 

The FAF images showed hyperFAF in the great majority of the cases, probably due to accumulation of lipofuscin and other fluorophores, as described by Spaide and Klancnik [7]. We found a much weaker correlation between a hypoFAF spot at the leakage site, as reported by Framme et al. [15], detected only in 3 eyes, comparing to 12 eyes with a hypoNIA spot in the same situation.

Ayata et al. [16] had similar findings in acute CSC, also presenting hypoNIA corresponding to the area of the serous retinal detachment and to the leakage point. Interestingly, he also found hypoNIA spots that were not leaking on angiogram, hypothesizing that the pathological site of the chorioretinal disturbance could be more extensive than expected. We had the opportunity to correlate the NIA images to OCT, and the hipoNIA spots, corresponding or not to leaking points, presented a PED or RPE focal irregularity, sustaining a choroidal etiology for CSC.

Almost 60% of NIA examinations showed diffuse hypoNIA, although almost 25% displayed hyperNIA. These results may represent different stages of the disease. We did not observe the minute defect described by Fujimoto et al. [17] in any of the cases, although we confirmed the relation between the PED and the leakage site, seen in more than 63% of PEDs. The presence of leakage in the PEDs spots could help explain the CSC recurrence, the persistence of retinal detachment and symptoms in some cases, as the PEDs are active, in terms of leakage and lower RPE absorption.

One of the most interesting findings of this study, was the correlation of the PED in the SD-OCT with the hypoNIA spot and the point of leakage in the fluorescein angiography (FA). Almost 60% of PEDs corresponded to leaking sites that corresponded to hypoNIA areas, and twelve of thirteen eyes that showed leakage had a hypoNIA spot exactly at the leaking spot. Although FA is still an important exam for the management of CSC, these findings may be useful in guiding the treatment, since OCT and NIA may provide the location of leakage points by means of a noninvasive, dye-free examination, providing safety and precision for the patient and the doctor, matching with the treatment safety pursuit, as we can expect from the subthreshold diode micropulse photocoagulation [18]. This is a retrospective, not controlled or randomized study, evaluating chronic CSC eyes. A larger sampled study, including acute cases, are warranted to confirm these preliminary results.

## Figures and Tables

**Table 1:** 

	FAF (%)			NIA(%)		
	hypoFAF	mixedFAF	hyperFAF	hypoNIA	mixedNIA	hyperNIA
Detached area (*N* = 10)	10	20	70	30	20	50
Leakage spot (*N* = 13)	23.1	×	76.9	92.3	×	7.7
Window defect area (*N* = 8)	12.5	12.5	75	62.5	32.5	0
Overall image (*N* = 17)	17.6	11.8	70.6	58.8	17.6	23.5
PED (*N* = 22)				63.6	×	36.4

## References

[1] Moschos M, Brouzas D, Koutsandrea C (2007). Assessment of central serous chorioretinopathy by optical coherence tomography and multifocal electroretinography. *Ophthalmologica*.

[2] Robertson DM (1986). Argon laser photocoagulation treatment in central serous chorioretinopathy. *Ophthalmology*.

[3] Chaine G, Haouat M, Menard-Molcard C (2001). Central serous chorioretinopathy and systemic steroid therapy. *Journal Francais d’Ophtalmologie*.

[4] Conrad R, Weber NF, Lehnert M, Holz FG, Liedtke R, Eter N (2007). Alexithymia and emotional distress in patients with central serous chorioretinopathy. *Psychosomatics*.

[5] Wang M, Munch IC, Hasler PW, Prünte C, Larsen M (2008). Central serous chorioretinopathy. *Acta Ophthalmologica*.

[6] Jalkh AE, Jabbour N, Avila MP (1984). Retinal pigment epithelium decompensation. I. Clinical features and natural course. *Ophthalmology*.

[7] Spaide RF, Klancnik JM (2005). Fundus autofluorescence and central serous chorioretinopathy. *Ophthalmology*.

[8] Wang M, Sander B, Lund-Andersen H, Larsen M (1999). Detection of shallow detachments in central serous chorioretinopathy. *Acta Ophthalmologica Scandinavica*.

[9] Eandi CM, Chung JE, Cardillo-Piccolino F, Spaide RF (2005). Optical coherence tomography in unilateral resolved central serous chorioretinopathy. *Retina*.

[10] Delori FC, Dorey CK, Staurenghi G, Arend O, Goger DG, Weiter JJ (1995). In vivo fluorescence of the ocular fundus exhibits retinal pigment epithelium lipofuscin characteristics. *Investigative Ophthalmology and Visual Science*.

[11] Von Ruckmann A, Fitzke FW, Bird AC (1995). Distribution of fundus autofluorescence with a scanning laser ophthalmoscope. *British Journal of Ophthalmology*.

[12] Eldred GE, Katz ML (1988). Fluorophores of the human retinal pigment epithelium: separation and spectral characterization. *Experimental Eye Research*.

[13] Keilhauer CN, Delori FC (2006). Near-infrared autofluorescence imaging of the fundus: visualization of ocular melanin. *Investigative Ophthalmology and Visual Science*.

[14] Kellner U, Kellner S, Weinitz S (2010). Fundus autofluorescence (488 nm) and near-infrared utofluorescence (787 nm) visualize different retinal pigment epithelium alterations in patients with age-related macular degeneration. *Retina*.

[15] Framme C, Walter A, Gabler B, Roider J, Sachs HG, Gabel VP (2005). Fundus autofluorescence in acute and chronic-recurrent central serous chorioretinopathy. *Acta Ophthalmologica Scandinavica*.

[16] Ayata A, Tatlipinar S, Kar T, Unal M, Ersanli D, Bilge AH (2009). Near-infrared and short-wavelength autofluorescence imaging in central serous chorioretinopathy. *British Journal of Ophthalmology*.

[17] Fujimoto H, Gomi F, Wakabayashi T, Sawa M, Tsujikawa M, Tano Y (2008). Morphologic changes in acute central serous chorioretinopathy evaluated by Fourier-Domain optical coherence tomography. *Ophthalmology*.

[18] Chen SN, Hwang JF, Tseng LF, Lin CJ (2008). Subthreshold diode micropulse photocoagulation for the treatment of chronic central serous chorioretinopathy with juxtafoveal leakage. *Ophthalmology*.

